# Early Administration of Anti–SARS-CoV-2 Monoclonal Antibodies Prevents Severe COVID-19 in Kidney Transplant Patients

**DOI:** 10.1016/j.ekir.2022.03.020

**Published:** 2022-03-26

**Authors:** Juliette Gueguen, Charlotte Colosio, Arnaud Del Bello, Anne Scemla, Yohan N’Guyen, Claire Rouzaud, Claudia Carvalho-Schneider, Gabriela Gautier Vargas, Pierre Tremolières, A. Jalal Eddine, Christophe Masset, Olivier Thaunat, Melchior Chabannes, Paulo Malvezzi, Pierre Pommerolle, Lionel Couzi, Nassim Kamar, Sophie Caillard, Philippe Gatault

**Affiliations:** 1 Service de Néphrologie-Hypertension Artérielle, Dialyses, Transplantation Rénale, CHRU de Tours, Tours, France; 2 Service de Transplantation Rénale, Hôpital Robert Debré, Reims, France; 3 Department of Nephrology and Organ Transplantation, Toulouse Rangueil University Hospital, Toulouse, France; 4 Départment de Néphrologie et Transplantation Rénale, Hôpital Necker Enfants Malades, Assistance Publique-Hôpitaux de Paris, Paris, France; 5 Service de Médecine Interne, Maladies Infectieuses et Immunologie Clinique, Hôpital Robert Debré, Reims, France; 6 Service de Médecine Infectieuse, Hôpital Necker Enfants Malades, Assistance Publique-Hôpitaux de Paris, Paris, France; 7 Service de Médecine Interne et Maladies Infectieuses, CHRU de Tours, Tours, France; 8 Department of Nephrology and Transplantation, Strasbourg University Hospital, Strasbourg, France; 9 Service de Transplantation Rénale, CHU Montpellier, Montpellier, France; 10 Service de Transplantation Rénale, Hôpital Foch, Paris, France; 11 Institut de Transplantation Urologie Néphrologie (ITUN), CHU Nantes, Nantes, France; 12 Service de Transplantation Rénale, Hôpitaux Civils, Lyon, France; 13 Service de Néphrologie, Dialyse et Transplantation, CHU Besançon, Besançon, France; 14 Service de Néphrologie, Hémodialyse, Aphérèses et Transplantation Rénale, CHU Grenoble-Alpes, Grenoble, France; 15 Service de Néphrologie et Transplantation Rénale, CHU Amiens Sud, Amiens, France; 16 Department of Nephrology, Transplantation, Dialysis and Apheresis, CHU Bordeaux, Bordeaux, France

**Keywords:** COVID-19, monoclonal antibody, transplantation, viral infection

## Abstract

**Introduction:**

Kidney transplant recipients (KTRs) are prone to develop severe COVID-19 and are less well protected by vaccine than immunocompetent subjects. Thus, the use of neutralizing anti–SARS-CoV-2 monoclonal antibody (MoAb) to confer a passive immunity appears attractive in KTRs.

**Methods:**

We performed a French nationwide study to compare COVID-19–related hospitalization, 30-day admission to intensive care unit (ICU), and 30-day death between KTRs who received an early infusion of MoAb (MoAb group) and KTRs who did not (control group). Controls were identified from the COVID-SFT registry (NCT04360707) using a propensity score matching with the following covariates: age, sex, delay between transplantation and infection, induction and maintenance immunosuppressive therapy, initial symptoms, and comorbidities.

**Results:**

A total of 80 KTRs received MoAb between February 2021 and June 2021. They were matched to 155 controls. COVID-19–related hospitalization, 30-day admission to ICU, and 30-day death were less frequently observed in the MoAb group (35.0% vs. 49.7%, *P* = 0.032; 2.5% vs. 15.5%, *P* = 0.002; 1.25% vs. 11.6%, *P* = 0.005, respectively). No patient required mechanical ventilation in the MoAb group. The number of patients to treat to prevent 1 death was 9.7.

**Conclusion:**

The early use of MoAb in KTRs with a mild form of COVID-19 largely improved outcomes in KTRs.

## Graphical abstract

**Figure:**
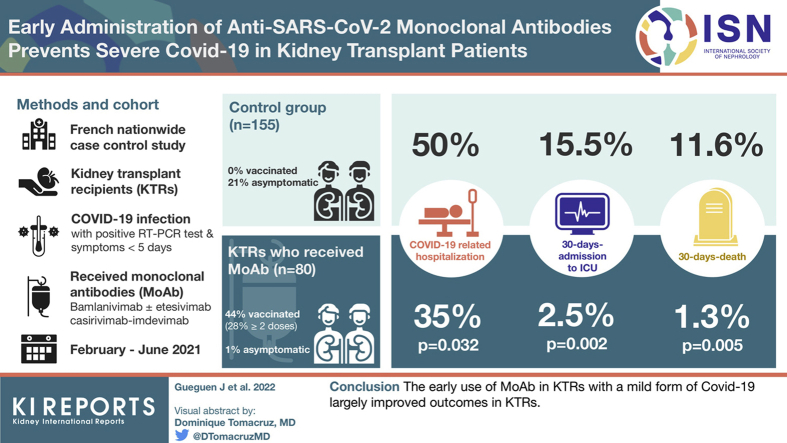

The high frequency of severe comorbidities in KTRs makes them very likely to develop severe COVID-19 caused by SARS-CoV-2.^1^, ^2^, ^3^ In addition, their immunization rates after vaccination are lower than in immunocompetent subjects after 1, 2, and even 3 injections of vaccines, with often a low level of anti–SARS-CoV-2 spike protein antibodies.^4^^,^^5^ Thus, a large proportion of KTRs remains currently exposed to the risk of severe COVID-19 despite vaccination.^6^ In this context, the use of neutralizing anti–SARS-CoV-2 MoAb to confer a passive immunity appears attractive in these patients.

Two large clinical trials have been conducted in high-risk outpatients with recent mild-to-moderate COVID-19. On the basis of a similar virologic primary outcome, both studies demonstrated that monoclonal therapeutic antibodies targeting receptor-binding domain accelerated the viral load decline.^7^, ^8^, ^9^ In addition, these 2 trials suggested that combinations of bamlanivimab-etesevimab and casirivimab-imdevimab would reduce the proportion of patients requiring further hospitalizations or visits for worsening COVID-19. Finally, US Food and Drug Administration and then European Medicines Agency delivered an emergency use authorization for those therapeutic antibodies in patients at risk of severe COVID-19, including KTRs.

So far, data about the use of these MoAbs in KTRs remain limited. Two small case series reported a good tolerance profile and a low rate of COVID-19–related hospitalization after an early infusion of bamlanivimab.^10^^,^^11^ Similar results were obtained in 25 solid organ transplant (SOT) recipients treated with casirivimab-imdevimab.^12^ Finally, Del Bello *et al.*^13^ compared 16 SOT recipients who received different MoAbs to 32 nonmatched hospitalized patients and found that neutralizing anti–SARS-CoV-2 MoAbs could prevent acute respiratory failure.

To investigate the efficacy of anti–SARS-Cov-2 MoAb to prevent severe COVID-19 in KTRs, we conducted a nationwide case-control study comparing outcomes between all KTRs in France who had received MoAb between March 2021 and June 2021 in France and matched KTRs issued from the French SOT COVID registry^1^ using the propensity score matching method. We compared their risk of COVID-19–related hospitalization, ICU admission, and death.

## Methods

### Patients

In France, the use of MoAb binding the SARS-CoV-2 spike protein was approved on February 2, 2021, for SARS-CoV-2–infected immunosuppressed patients. Eligibility criteria for MoAb infusion were as follows: (i) positive result of reverse transcriptase–polymerase chain reaction test performed on nasopharyngeal swab specimens; (ii) recent symptoms (≤5 days); and (iii) no need of oxygen. We included all KTRs who received MoAb between February and June 15, 2021, in France.

**Figure 1:**
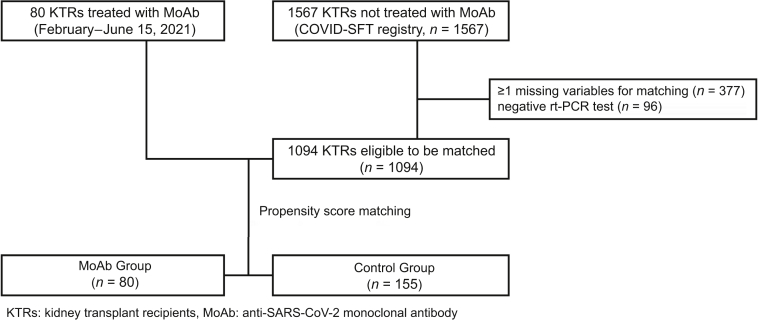
Flowchart. KTR, kidney transplant recipient; MoAb, monoclonal antibody; rt-PCR, real-time polymerase chain reaction.

Control group was issued from the French SOT COVID registry (NCT04360707, ethical approval by the Institutional Review Board of the Strasbourg University) that enrolled 1567 KTRs from 31 centers who have COVID-19 until December 31, 2020. The patients with missing data for 1 of the variables needed for matching (*n =* 377) and those with a negative real-time polymerase chain reaction test result (*n* = 96) were excluded from the study (Figure 1).

### Data

Data were collected retrospectively using hospital databases and clinical records for all patients. Each medical record was individually and manually reviewed by a medical doctor in each center. Data collection included patient (age, sex, smoking status, comorbidities: diabetes mellitus, body mass index, hypertension, cardiopathy, chronic lung disease, kidney function) and transplant (induction and maintenance immunosuppressive therapy, donor type, rank) characteristics and COVID-19 characteristics and outcomes (delay between transplantation and COVID-19, symptoms, hospitalization, ICU admission, death).

### Outcomes

Primary analyses included COVID-19–related hospitalization, ICU admission, and death rates for the first 30 days after the diagnosis. COVID-19–related hospitalization was defined as hospitalization needed because of the severity of the symptoms. For this reason, we did not consider hospitalization for MoAb infusion (as initially recommended by the French authorities) and nosocomial COVID-19 if patients did not show any exacerbation during hospitalization. We secondarily analyzed rate of severe COVID-19 defined as admission (or transfer) to ICU, need for mechanical ventilation, or death. We finally provided 3-month patient survival defined as the time between the date of positive real-time polymerase chain reaction test and the date of death or last follow-up.

### Propensity Score Matching

KTRs in the MoAb group were matched to KTRs issued from COVID-SFT registry by a propensity score, using a 1:2 ratio, nearest neighbor method, and a caliper of 0.3 with the following covariates: age, sex, time between transplant and positive SARS-CoV-2 real-time polymerase chain reaction test result, induction therapy, maintenance immunosuppression; body mass index, diabetes mellitus, history of cardiovascular disease, hypertension, chronic lung disease and creatinine, fever and dyspnea at time of MoAb infusion (or COVID-19 diagnosis). The balance of covariate distribution between the 2 groups was assessed using standardized mean differences (Supplementary Data).

### Missing Data

Propensity score matching method requires fully completed data. To keep all KTRs included in the study, we imputed the few missing data in the MoAb group as follows: 3 data were missing for induction and were imputed by “basiliximab” to allow matching with basiliximab-treated controls to favor the control group; 1 data were missing for body mass index and imputed using the median of body mass index for the same age and sex. Patients with missing data in the control group were excluded before matching.

### Statistical Analysis

Continuous variables were described using medians and interquartile ranges and compared using the Mann-Whitney rank-sum test. Categorical variables were described using proportions and compared using Fisher exact test. Survivals were represented using Kaplan–Meier curves and compared using a 2-sided log-rank test. Statistical analyses were performed using Stata (StataCorp College Station, TX) and R Studio (RStudio, version 1.2.1335). All statistical tests were 2-sided, and *P* < 0.05 was considered as significant.

## Results

### Patient Characteristics

**Figure 2:**
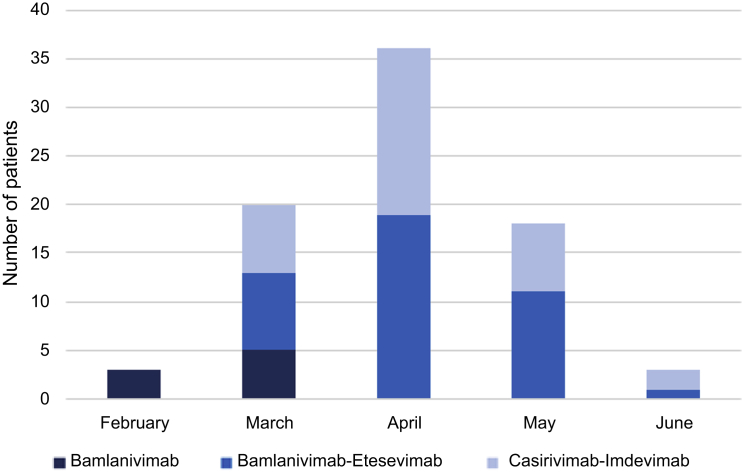
Use of anti–SARS-Cov-2 therapeutic antibodies between February 2021 and June 2021. The number of patients treated with bamlanivimab as monotherapy (*n =* 8), bamlanivimab-etesevimab (*n =* 39), or casirivimab-imdevimab (*n =* 33) is represented by month.

Overall, 80 KTRs from 13 transplant centers received MoAb. They were initially treated with bamlanivimab (*n =* 8) and then with bamlanivimab-etesevimab (*n =* 39) or casirivimab-imdevimab (*n =* 33) (Figure 2). The mean delay between the first symptoms and the administration of MoAb was 3.8 ± 2.4 days with 6 patients treated beyond the fifth day.

The matching process identified 155 control patients among 1094 patients included from the COVID-SFT registry. Their basal characteristics were compared with those of patients treated with MoAb (Table 1). As expected, no significant differences were observed regarding age, immunosuppressive regimen, delay of transplantation, and comorbidities. Of note, the proportion of asymptomatic KTRs was lower in the MoAb group than in the control group (1.3% vs. 21.3%, *P* < 0.001).

**Table 1:** Patient characteristics

Variable	MoAb group (*n =* 80)	Control group (*n =* 155)	*P* value
Male sex, *n* (%)	45 (56.3)	90 (58)	0.889
Age, median (IQR)	57 (46–64.5)	57 (44–65)	0.940
Age >60 yr, *n* (%)	34 (43)	65 (42)	0.999
BMI (kg/m^2^), median (IQR)	26.1 (23.7–30.1)	27 (23.4–30)	0.952
Time from transplantation to COVID-19 (mo), median (IQR)	57 (17–139)	64 (21–140)	0.521
COVID-19 during the first post-transplant yr, *n* (%)	15 (18.8)	26 (16.8)	0.719
First transplantation, *n* (%)	67 (83.8)	142 (91.6)	0.081
Combined transplantation, *n* (%)	4 (5)	6 (3.9)	0.999
Living donor, *n* (%)	15 (18.8)	23 (14.8)	0.458
T-cell depletion, *n* (%)	42 (52.5)	87 (56.1)	0.776
Comorbidities, *n* (%)			
Diabetes mellitus	27 (33.8)	50 (32.3)	0.884
Cardiac disease	21 (26.3)	38 (24.5)	0.874
Hypertension	62 (77.5)	127 (81.9)	0.488
Cancer	10 (12.5)	26 (16.8)	0.448
Respiratory disease	11 (13.8)	24 (15.5)	0.847
BMI ≥ 30 kg/m^2^	20 (25)	42 (27.1)	0.757
Number	1.9 ± 1.3	2.0 ± 1.2	0.688
Tobacco use, *n* (%)	8 (10)	18 (11.6)	0.423
Symptoms, *n* (%)			
None	1 (1.3)	31 (21.3)	**<0.001**
Dyspnea	11 (13.8)	28 (18.1)	0.462
Fever	34 (42.5)	57 (36.8)	0.400
Headache	22 (27.5)	29 (18.7)	0.135
Myalgia	26 (32.5)	28 (18.9)	0.033
Cough	45 (56.3)	70 (45.2)	0.130
Diarrhea	20 (25)	45 (29)	0.542
Vomiting	3 (3.8)	9 (6.2)	0.546
Immunosuppression, *n* (%)			
Calcineurin inhibitor	71 (88.8)	141 (91)	0.645
Mycophenolic acid	60 (75)	110 (71)	0.542
Azathioprine	4 (5)	13 (8.4)	0.432
mTOR inhibitor	13 (16.3)	26 (16.8)	0.999
Belatacept	4 (5)	3 (1.9)	0.233
Steroids	62 (77.5)	119 (76.8)	0.999
Suspension of immunosuppression, *n* (%)			
Calcineurin inhibitor	4 (5.6)	18 (13.5)	0.083
Mycophenolic acid/azathioprine	27 (42.2)	47 (38.2)	0.598
mTOR inhibitor	6 (46.2)	14 (53.9)	0.651
Vaccinated	33 (44.2)	0 (0.0)	**<0.0001**
Creatinine level at baseline before COVID-19 (μmol/l), med (IQR)	125 (100–165)	130 (100–174)	0.699

BMI, body mass index, IQR, interquartile range, MoAb, monoclonal antibody; mTOR, mammalian target of rapamycin.

Bold data indicate significant difference.

Regarding immunosuppressive therapy management after diagnosis of COVID-19, we found that a slight majority of KTRs (*n =* 126, 53.6%) underwent a reduction of their immunosuppression (MoAb: 39 [48.8%] vs. controls: 87 [58.0%], *P* = 0.179). In detail, clinicians suspended mycophenolic acid, calcineurin inhibitor, or mammalian target of rapamycin inhibitors in 39.6%, 10.8%, and 51.3% of patients in similar proportion in both groups. Treatment with belatacept was maintained in all treated KTRs (*n =* 7).

Finally, the unique clinically relevant difference between the groups concerned vaccination. Indeed, because vaccination was not yet available in 2020, no patient in the control group was vaccinated. In contrast, 33 KTRs (44.2%) treated with MoAb had received at least 1 dose of vaccine (1 dose: 17, 2 doses: 19, 3 doses: 3). Antispike protein antibodies before MoAb injection were detected in only 10 patients.

### Patient Outcomes

In the first month, KTRs were less frequently hospitalized for worsening COVID-19 in the MoAb group than in the control group (28 [35.0%] vs. 77 [49.7%], *P* = 0.032). Of note, 15 of 28 patients (53.6%) were vaccinated (1 dose: 8, 2 doses: 6, 3 doses: 1), 3 of whom had detectable antispike antibodies before injection of MoAb. Likewise, we observed a lower proportion of patients admitted to the ICU in the MoAb group (2 [2.5%] vs. 24 [15.5%], *P* = 0.002). Both patients admitted to the ICU in the MoAb group never required mechanical ventilation (Table 2).

**Table 2:** Comparison of outcomes at 1 month of kidney transplant recipients treated or not with anti–SARS-CoV-2 antibodies

Outcomes	MoAb group (*n =* 80)	Control group (*n =* 155)	*P* value
Severe COVID-19, *n* (%)	3 (3.8)	30 (19.4)	0.001
Admission to ICU, *n* (%)	2 (2.5)	24 (15.5)	0.002
Need for mechanical ventilation, *n* (%)	0 (0.0)	18 (11.6)	<0.001
Death, *n* (%)	1 (1.25)	18 (11.6)	0.005

ICU, intensive care unit.

Only 1 death (1.25%) occurred after administration of MoAb. It happened in a 76-year-old male patient with numerous comorbidities and advanced vascular dementia disqualified for ICU admission. COVID-19 was diagnosed 10 years after the transplantation while the patient had already been hospitalized for confusion (SARS-CoV-2 negative result on admission) in the context of a cluster recently developed in the unit. The early injection of a bamlanivimab-etesevimab combination 1 day after the first symptoms did not prevent the worsening of COVID-19 with a severe respiratory disease leading to death 5 days later.

**Figure 3:**
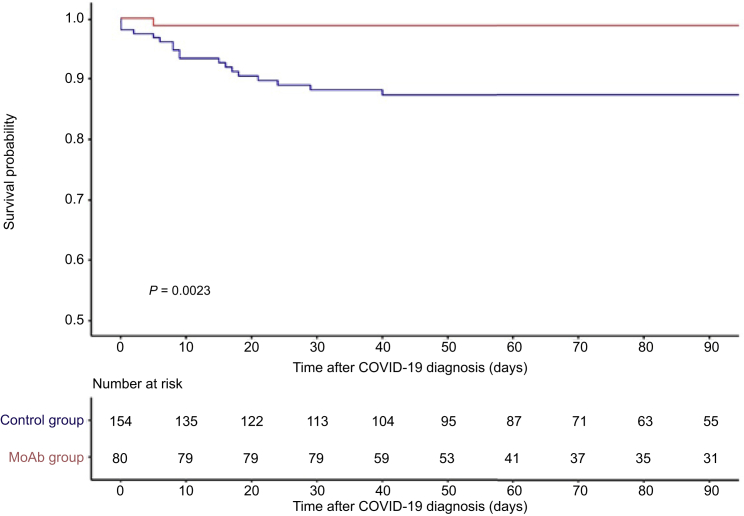
The 90-day patient survival in the patients treated with an early injection of anti–SARS-Cov-2 therapeutic antibody was greater than and matched than that of control recipients. Patients treated with therapeutic antibodies and control patients are presented by continuous and dotted line, respectively. The survival was higher in the patients treated with monoclonal antibodies (log-rank, *P* = 0.004). MoAb, monoclonal antibody.

The mortality in the control group was higher with 21 deaths (13.6%) notified during the follow-up (Figure 3). Of 21 deaths, 18 (85.7%) occurred in the first month. The 30-day mortality rate was lower in the MoAb group (*P* = 0.008), as shown in Table 2. On the basis of an absolute risk reduction of 10.35%, we estimated that 9.7 patients should be treated to avoid 1 death.

## Discussion

In this carefully designed, multicenter, case-control study, we investigated the effectiveness of early infusion of neutralizing MoAb in the treatment of mild or moderate COVID-19 in KTRs. We demonstrate that an early infusion of MoAb reduces the risk of severe COVID-19 with a 10-fold lower risk of admission to ICUs and death and, therefore, strongly comforts preliminary results reported in SOT recipients without major side effects.^11^, ^12^, ^13^

This remarkable effectiveness of MoAb is the result of the strong potency of antispike MoAb to limit the spread of the virus at the first stage of the infection. As most of the neutralizing antibodies in SARS-CoV-2–infected patients which contribute to the protection of the patients, currently available MoAbs recognize the receptor-binding domain located on the S1 subunit of viral spike, which is the main antigenic domain with the N-terminal domain.^14^ Because these domains are less conserved than other parts of the spike protein, SARS-CoV-2 variants can escape to MoAb neutralization.^15^^,^^16^ It occurred for bamlanivimab unable to neutralize the now dominant SARS-CoV-2 variant B.1.617 and therefore revoked as monotherapy. In our study, patients in the MoAb group were mostly exposed to B.1.1.7 variant considered as more pathogenic than ancestor Wuhan strain still dominant when control patients were infected, because the period of enrollment differed between the 2 groups.^17^^,^^18^ Although MoAb could be more broadly used as preventive therapy in nonresponder to vaccination or in prolonged COVID-19,^19^^,^^20^ it is important to keep in mind that effectiveness of these treatment must regularly be updated^21^ and therapeutic strategies potentially refined taking account the geographic distribution of different SARS-CoV-2 strains. The last illustration is the recent propagation of the Omicron variant that escapes to casirivimab and imdevimab. The use of antibodies that target highly conserved epitopes, such as sotrovimab, could represent an attractive alternative.^22^, ^23^, ^24^

It is important to consider the efficacy of MoAb in light of risk profile of the population. The 2 first sponsored clinical trials were conducted in immunocompetent patients supposed to be at high risk of worsening COVID-19. The clinical benefit observed in patients treated with bamlanivimab-etesevimab and casirivimab-imdevimab remained uncertain because of the unexpected low number of hospitalization.^7^^,^^9^ Several observational studies brought more convincing results with a lower risk of hospitalization in patients treated with MoAb.^25^^,^^26^ However, one of the studies that compared 594 patients with 5536 unmatched contemporaneous patients and 7404 historical patients failed to show a clear effect on mortality.^26^ This was likely due to the short follow-up (14 days) and again the fairly low risk of death in control patients (1%). In our study, 11.6% of matched KTRs passed away during the first month, close to the proportion found in a well-designed case-control study that found a strong reduction of mortality using MoAb (0% vs. 9.4%).^25^ It is of note that real-life studies that included a large number of immunocompromised patients unfortunately did not discriminate SOT from other causes of immunosuppression (hematopoietic stem cell transplant recipient, HIV, or currently receiving chemotherapy) and did not specifically investigate the efficacy of MoAb in immunocompromised patients.^26^^,^^27^ Finally, we can conclude that immunosuppression confers a high risk of worsening COVID-19 but does not alter the efficacy of MoAb.

Today, the first-line weapon against COVID-19 is obviously vaccines, especially mRNA-based vaccines.^28^, ^29^, ^30^ Nowadays, it is clear that immune response to COVID-19 vaccination is affected by immunosuppressive drugs. Indeed, although mRNA-based vaccine elicits antibody response in >95% in general population, the proportion of SOT recipients who developed antispike protein after 1 dose is meager, estimated between 5% and 17%.^4^^,^^31^^,^^32^ Second and third injections increase the proportion of SOT recipients with antispike protein antibodies to approximately 40% and 70%,^32^, ^33^, ^34^ but not in KTRs treated with belatacept who keep a very weak immune response.^35^ In our study, only 10 patients (12.5%) were sensitized before infusion of MoAb. In our opinion, a positive impact of vaccination cannot be considered as sufficient to explain the drastic reduction of worsening COVID-19 in KTRs treated with MoAb, even though complementary data about neutralizing antibodies would have been of interest. Because the positive effect of MoAb is restricted to seronegative patients,^7^ a systematic quantification of antispike or anti-RBD antibodies after the third dose or the booster dose should be recommended to select patients who would benefit an early MoAb injection. Our study in addition underscores that caregivers should provide consistent information about the importance to promptly diagnose COVID-19 if they face any suggestive symptoms.

In conclusion, the early use of MoAb in KTRs with a mild form of COVID-19 dramatically improves outcome of KTRs. Our results highlight the importance of a prompt diagnosis after first symptoms (or exposure to the virus) among SOT recipients who are frequently nonresponders to vaccines, alongside the maintenance of nonmedical preventive measures.

## Disclosure

All the authors declared no competing interests.

## Supplementary Material

Supplementary File (PDF)

**Loveplot for balance of covariances between KTRs in MoAb group and control group before and after adjustment.**

**STROBE statement.**
